# EGCG attenuated spinal cord injury by inhibiting ferroptosis via activation of HMOX1 expression and suppression of HIF-1 signaling pathway

**DOI:** 10.22038/ijbms.2025.82651.17864

**Published:** 2025

**Authors:** Han Yang, Fei Ye, Liuxu Chen, Linyu Yang, Jianping Kang

**Affiliations:** Department of Orthopaedics, The Affiliated Hospital of Southwest Medical University, Luzhou 646000, P.R. China

**Keywords:** Epigallocatechin gallate, Ferroptosis, HIF-1, HMOX1, Spinal cord injury

## Abstract

**Objective(s):**

Epigallocatechin gallate (EGCG) exhibits various biological effects, including antiviral, anti-inflammatory, cardioprotective, and lipid-regulating properties. This study aims to investigate the therapeutic effects and mechanisms of EGCG in spinal cord injury (SCI).

**Materials and Methods:**

The bioinformatic databases were used to screen therapeutic target genes for drugs against SCI. Component-Target-Disease networks were constructed with Cytoscape software, and inter-target interactions were analyzed using the String database. Additionally, KEGG pathway enrichment analyses were conducted on the identified target genes. SCI was evaluated by detecting inflammation-related factors, H&E staining, and immunohistochemistry. Furthermore, ROS and JC1 staining were performed on HT22 cells subjected to various treatments. Molecular mechanisms were investigated using western blot and qRT-PCR analyses.

**Results:**

Forty-four overlapping genes were identified as potential targets, with HMOX1, GPX-4, and HIF-1A emerging as central hub genes. Key pathways associated with these targets included Ferroptosis and HIF-1 signaling. *In vivo* studies demonstrated that EGCG effectively promotes motor function recovery and reduces the expression of proteins and genes such as IL-1β, IL-6, HIF-1α, and 4HNE.* In vitro* experiments showed that EGCG decreases ROS and intracellular lipid ROS levels in HT22 cells while increasing GPX-4 and HMOX1 expression to inhibit ferroptosis and HIF-1 signaling pathways.

**Conclusion:**

Our findings reveal a significant new mechanism by which EGCG can reduce SCI through the inhibition of ferroptosis, facilitated by the activation of HMOX1 expression and the down-regulation of the HIF-1 signaling pathway. This suggests its potential as a therapeutic option for this condition.

## Introduction

Spinal cord injury (SCI) is a disabling condition that seriously affects the quality of life of patients, imposes significant burdens on both patients and society, and has long been a prominent and challenging topic in clinical research. SCI is a type of external severe spinal cord disease caused by injuries, hematomas, infections, and tumors. The primary manifestation is the partial or complete loss of sensory and motor function in the limbs below the damaged segment (1, 2). In recent years, the incidence of SCI has been rising steadily, with reports indicating 10 to 83 new cases per million people worldwide each year. In descending order, the most common causes of SCI are traffic accidents, falls, and violent injuries (3). The incidence of SCI in China is very high, primarily due to vehicle collisions and high-altitude falls. Although extensive research has been conducted on spinal cord injury (SCI) in recent years, the pathogenesis remains not fully understood. As a result, clinical treatments for SCI are often not ideal, and the prognosis can be poor (4).

Traditional Chinese medicine (TCM) treatment has been a common treatment for late-stage SCI in recent years (5). TCM promotes spinal cord injury recovery by enhancing immune function and microcirculation. Its mechanisms include inhibiting inflammatory response, promoting the secretion of neurotrophic factors, inhibiting lipid peroxidation, improving microcirculation, inhibiting cell apoptosis, and promoting nerve cell regeneration (6). Many TCMs are used to treat spinal cord injury, including drugs rich in bioactive ingredients, such as *Gentiana macrophylla, Ligusticum chuanxiong, and Astragalus membranaceus *(7). These TCMs have multiple effects, such as anti-inflammatory, anti-oxidant, and anti-thrombotic, and can help repair damaged spinal cord tissue.

EGCG is a natural polyphenol found primarily in green tea, and it has been widely studied for its diverse biological effects (8). Experiments confirmed that H₂O₂ was used to stimulate PC-12 cells to establish a typical oxidative stress nerve damage cell model. It was found that different doses of EGCG applied to PC-12 cells could enhance H₂O₂-induced cell viability, block nuclear fragmentation and DNA agglutination, and significantly improve the functional state of the cells (9). Studies reported that after intraperitoneal injection of different doses of EGCG into ApoE⁻/⁻ mice, the serum concentrations of related factors, such as the inflammatory factors IL-6, TNF-α, and the chemokine MCP-1, were reduced. Immunofluorescence detection also showed that EGCG could reduce the infiltration of inflammatory cells in the aortic arch of the mice (10, 11). Thus, investigating the pharmacological effects of EGCG holds significant potential for advancing clinical drug development.

In this study, bioinformatics techniques were utilized to identify therapeutic target genes for EGCG in the context of SCI. Functional analyses, including KEGG pathway enrichment, were conducted to uncover potential signaling pathways involved. The therapeutic efficacy of EGCG was validated using an SCI mouse model, while a cell model was employed to investigate the underlying mechanisms further. Additionally, gene and protein expression levels associated with the identified signaling pathways were analyzed to clarify the role of EGCG in SCI treatment. These findings offer valuable insights and establish an experimental foundation for the clinical application of EGCG in SCI therapy.

## Materials and Methods

### Experimental animals

**Figure 1:**
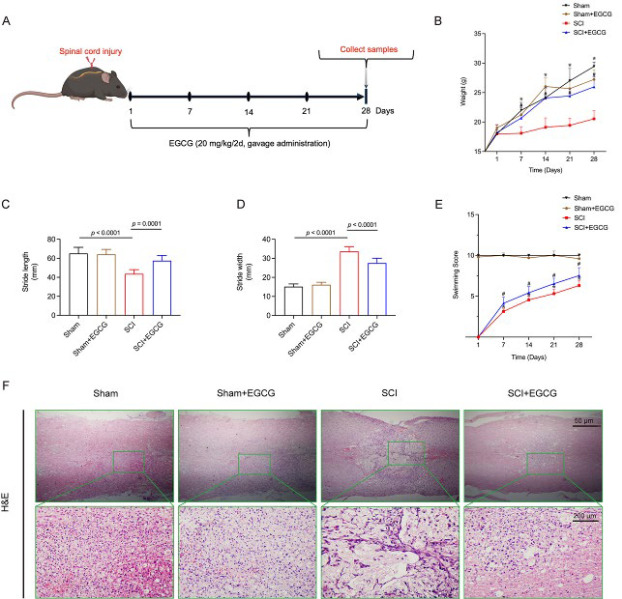
EGCG reduces spinal cord injury in SCI mice

Robust, specific-pathogen-free female C57BL/6J mice, 8 weeks old and weighing 18–20 g, were from the Animal Research Institute of Southwest Medical University. All animal experiments were approved by the Medical Ethics Committee of the Affiliated Hospital of Southwest Medical University (KY2023087). The spinal cord injury surgery was performed following the procedures described in previous studies (12). Briefly, a laminectomy was performed at the T9 level to expose the spinal cord. No. 5 Dumont forceps (Fine Science Tools), secured to a stereotaxic apparatus, were used to crush the spinal cord for 3 sec. The day of surgery is considered the first day of SCI; Figure 1A illustrates the flow chart of the animal study. Mice were randomly divided into four groups: (1) Sham group (n = 8), where mice underwent only laminectomy; (2) Sham + EGCG group (n = 8), where mice received gastric administration of EGCG; (3) SCI group (n = 8); (4) SCI + EGCG group (n = 8), where mice with SCI received gastric administration of EGCG. EGCG treatment began four hours post-SCI at a dose of 20 mg/kg, administered every other day until the 28th day. All mice were sacrificed on the 28^th^ day (chronic phase of SCI), and relevant experimental indices were assessed according to established protocols.

### Cell culture

*In vitro* experiments were conducted to shed light on how neuronal cells participate in the inflammatory response, promote nerve regeneration, or reduce ferroptosis. HT22 cell lines were obtained from Procell Life Science and Technology Co. (Wuhan, China) and cultured under standard conditions in a humidified incubator with DMEM supplemented with 10 % fetal bovine serum, 1 % penicillin, and streptomycin at 37 °C with 5 % CO₂. The culture medium was replaced every two days. When cell confluence reached 80–90 %, the cells were digested and subcultured. During the logarithmic growth phase, we used a cell counter to measure the current cell density, adjusted the cell concentration to 3 × 10⁵ cells/ml according to the formula (Dilution factor = Current Density / 3 × 10⁵) and seeded into large culture dishes to allow adherence. After removing the initial medium and washing twice with PBS, fresh medium was added. The cells were then divided into the following groups: Control group, LPS group (3 nmol/l lipopolysaccharide), LPS + EGCG group (3 μmol/l EGCG), LPS + CAY10585 group (30 μM CAY10585), and LPS + Fer-1 group (1 μM Fer-1).

### Behavioral tests

A footprint analysis was performed on day 28 post-injury, following the protocol of previous literature (13). Mice had their forelimbs dipped in red dye and their hindlimbs in blue dye. Non-toxic, water-soluble ink was used on clean paper, and mice could walk across a paper-lined pathway to capture their footprints. The resulting footprints were analyzed for parameters like stride length and step width to evaluate the recovery of locomotor function. Multiple trials were carried out to ensure data reliability.

We employed the swimming score method on day 28 post-injury to evaluate locomotor performance. Briefly, mice were allowed to swim in a 1-meter-long, 6-centimeter-wide tank with an island at the end where they could climb out of the water. We assessed swimming performance by scoring several aspects of their movement, including hindlimb motion, coordination between the hindlimbs and forelimbs, tail position, paw placement, and both sagittal and coronal balance. Each mouse was required to cross the tank twice, and scores were assigned based on their performance in each session (14, 15). The assessment involved several key components: hindlimb movement was scored from 0 to 5 points, hindlimb/forelimb coordination was rated from 0 to 2 points, tail position was assessed with a score range of 0 to 1 point, paw positioning was also rated from 0 to 1 point, and sagittal and coronal balance were evaluated with scores ranging from 0 to 1 point.

### Bioinformatic analysis

In this study, we used the TCMSP database to identify the chemical components and targets of EGCG, applying criteria of oral bioavailability (OB) ≥ 30 % and drug-likeness (DL) ≥ 0.18. After identifying these targets, we searched for their corresponding genes across various databases. We used ‘spinal cord injury’ as a keyword to find targets associated with SCI and extracted relevant targets from GeneCards and Disgenet. Target genes were gathered from both databases, and duplicates were eliminated to isolate disease-specific target genes (16). Additionally, relevant target genes for a mouse model of SCI were obtained from the GEO database (GSE92657) (17). Venn diagrams were created to identify overlapping genes by intersecting EGCG target genes, therapeutic model expression genes for EGCG, and disease-specific target genes. The common genes from the drug-disease intersections were then uploaded to the STRING database to construct protein-protein interaction (PPI) networks. The composite score was set to a high-confidence threshold (≥ 0.9) to ensure reliability. The resulting PPI network data were imported into Cytoscape, where the degree of each network node was calculated. Using cytoHubba, subnetworks were extracted for the top 15 nodes with the highest degree, highlighting their central role in the network. KEGG pathway enrichment analysis was conducted using the DAVID Functional Annotation Chart tool (Version 6.8) (18, 19).

### Histology analysis

The spinal cord tissues were fixed in 10% neutral formalin solution, rinsed with deionized water, and then dehydrated in 70%, 80%, 95%, and 100% ethanol step by step (1.5 hr per step). Xylene was used for 15 min to make the tissues transparent, and the tissues were embedded in paraffin after three hours of waxing. Paraffin-embedded spinal cord tissues were sectioned into 4 μm slices. The sections were soaked in xylene I and II for clearing and then dehydrated through a graded alcohol series. Following a 3-5 min soak in PBS, the nucleus was stained with hematoxylin and the cytoplasm with eosin. After the slides were dried and mounted with neutral resin, the desired images were selected and captured under a microscope using a computer-assisted color image analysis system (Leica Microscope DM-2700M, Germany).

### ELISA analysis

Spinal cord tissue samples were collected for IL-1β and IL-6 analysis. Fix the antigen or antibody in the wells of the microplate and use the blocking buffer to prevent nonspecific binding. The sample or standard to be tested was added to make the antigen-antibody reaction sufficient. A washing step was performed to remove unbound material and reduce background noise. An enzyme-labeled detection antibody is added, and it binds to a specific antigen or antibody. With the addition of the substrate, the enzyme-catalyzed reaction generates a measurable signal, usually a chromogenic reaction. Signal intensity was measured using a microplate reader. The concentration of specific antigens or antibodies in the sample was calculated from the absorbance values and the standard curve.

### Iron measurements

The spinal cord tissues were homogenized in PBS to measure iron levels, and the supernatant was collected after centrifugation. Iron concentrations in the samples were assessed using the Iron Assay Kit (ab83366, Abcam), following the manufacturer’s instructions (20).

### GPX-4 activity and GSH level measurements

For GPX-4 activity and GSH levels, GPX-4 activity was measured with phosphatidylcholine hydroperoxide as the substrate following the previously described method. The total GSH levels in mouse spinal cord tissue were quantified using the GSH/GSSG Assay Kit from Beyotime, Shanghai, China.

### Immunohistochemistry

After dehydration, dewaxing, and other preparatory steps, the spinal cord tissue was embedded in paraffin and sectioned into 4 mm slices. The sections were soaked in xylene I and II for clearing and then dehydrated through a graded alcohol series. Following a 3-5 min soak in PBS, the sections were treated with H₂O₂ for 10 min and heated in a water bath with 0.01 mol/L sodium citrate buffer for 25 min. The sections were then incubated overnight at 4 °C with the primary antibody of GPX-4 and HMOX1 (Cat NO. A25009; A1346, dilution 1:100), followed by incubation with the secondary antibody (Cat NO. ZB-2306, Zhongshan Golden Bridge, Beijing, dilution 1:1000) at room temperature for two hours. DAB and hematoxylin were used for staining. The desired images were captured under a microscope, and the brown-stained areas in each image were quantified using Image J software.

### Mitochondrial membrane potential (Δψm) analysis

JC-1 staining was used to assess mitochondrial membrane potential after 24 hr of treatment. The cells were washed twice with PBS and then incubated with the JC-1 staining solution in a 37 °C cell incubator for 20 min. After incubation, the supernatant was removed, and the cells were rinsed twice with the JC-1 buffer solution. The slides were then mounted with an anti-fade mounting medium. Images were captured using an upright fluorescence microscope. JC-1 staining typically exhibits green fluorescence in cells with disrupted membrane potential, while normal cells should display red fluorescence.

### ROS and Lipid ROS measurements

HT22 cells were stained with the DCFH-DA fluorescent probe to measure intracellular ROS levels. The cells were rinsed with pre-warmed PBS at 37 °C, then incubated with 10 μM DCFH-DA (1:1000, serum-free) at 37 °C for 20 min. Following incubation, the cells were washed thrice with serum-free DMEM at 37 °C. Micrographs of 10 fields of view for each of the three independent experiments were captured at 630x magnification using a Leica confocal microscope. The fluorescence intensity of DCFH-DA (green) was quantified using the ImageJ software package.

Cells were washed twice with PBS, and the staining solution was added at 1 to 10 μM concentrations. The cells were then incubated for 30 min in a 37 °C cell culture incubator. After incubation, the dye was removed, and the cells were washed three times with PBS and sealed with an anti-fade mounting medium. The images were observed using an upright fluorescence microscope. In its reduced state, Bodipy 581/591 C11 displays red fluorescence with an excitation/emission maximum of 581/591 nm. After oxidation, the excitation/emission maximum shifts to 488/510 nm, resulting in green fluorescence.

### Western blot analysis

Extract proteins from spinal cord tissue or HT22 using RIPA buffer with protease and phosphatase inhibitors. Perform SDS-PAGE to separate the proteins, then transfer them to a PVDF membrane. Block the membrane with a 5 % milk-free solution to prevent nonspecific binding, and wash it with TBST to remove any non-specifically bound material. Incubate the membrane with primary antibodies of GPX-4, HMOX1, ACSL-4, and HIF-1a (Cat NO. A25009; A1346; A20414; A11945, ABclonal Technology, Wuhan, China) overnight at 4 °C, and then amplify the detection signal using a secondary antibody (Cat NO. AS003, AS029, ABclonal Technology, Wuhan, China) coupled with horseradish peroxidase (HRP) at 37 °C for 1.5 hr. Detect the protein signal on the membrane with a high-sensitivity chemiluminescence kit, such as Millipore, which works by HRP catalyzing the substrate to produce a visible light signal revealing the protein bands. Finally, advanced image processing software like Image J can be used for accurate quantitative analysis of the protein bands to determine the expression levels of the target proteins.

### Real-time PCR analysis

Quantitative reverse transcriptase polymerase chain reaction (qRT-PCR) is employed to measure mRNA levels of specific genes. The process starts with RNA extraction using a specialized kit, followed by reverse transcription to convert RNA into cDNA. This reverse transcription is typically conducted at 50 °C for 15–60 min, after which the enzyme is deactivated. The synthesized cDNA is then diluted for use in qPCR, a type of quantitative PCR. The primer sequences used in the present study were as follows: HIF-1α forward: 5′-CTGCCACTGCCACCACAACTG-3′, reverse: 5′-TGCCACTGTATGCTGATGCCTTA G-3′; 4HNE forward: 5′-TGCCGTAATGCCTACTTAGTGCTG-3′, reverse: 5′- CTCCCG ACAGGATCTTTACG-3′; GAPDH forward: 5′-GAAGGAATGGGTCGGAGTC-3′, reverse: 5′-GAAGATTGGGATGGGATTTC-3′. The qPCR reactions are performed through thermal cycling, which amplifies the DNA. After amplification, qPCR curves are generated, and each sample’s cycle threshold (CT) values are recorded. The relative expression levels of the target genes are determined using the ΔΔ CT method, with internal controls serving as references.

### Statistical analysis

The number of independent repetitions of all experiments reached at least three. All data were expressed as mean value ± standard error of the mean (SEM). Statistical analysis was performed by one-way ANOVA, followed by the Bonferroni multiple comparison test using GraphPad Prism (version 9.0). A value of *P<*0.05 was considered statistically significant.

## Results

### EGCG promotes motor function recovery in SCI animal model

To evaluate the potential therapeutic effects of EGCG on functional recovery following SCI, we developed an experimental mouse SCI model. On the 28^th^ day of modeling and treatment, motor functional indicators were tested independently. Compared with the Sham group, body weight in the SCI group decreased significantly. However, there was no significant difference in body weight between the Sham and the Sham + EGCG groups. In contrast, body weight in the SCI + EGCG group increased significantly (Figure 1B). Motor function detections show that EGCG treatment enhanced hindlimb function in SCI mice. Mice in the SCI + EGCG group exhibited longer stride lengths and narrower step widths than those in the SCI group (Figure 1C-D). Likewise, in the swimming score test, SCI mice treated with EGCG exhibited a significant improvement in motor function (Figure 1E). H&E staining revealed that EGCG treatment significantly reduced the area of spinal cord damage compared to untreated SCI mice, with less loss of white matter and central gray matter. Additionally, EGCG ameliorated nerve fiber disorganization and reduced necrosis of nerve cells compared to the SCI group. These findings indicate that EGCG significantly improved the recovery of hindlimb motor function in mice following SCI (Figure 1F).

### Bioinformatics analysis reveals significant down-regulation of HMOX1 and activation of the HIF-1 signaling pathway in SCI mice

**Figure 2:**
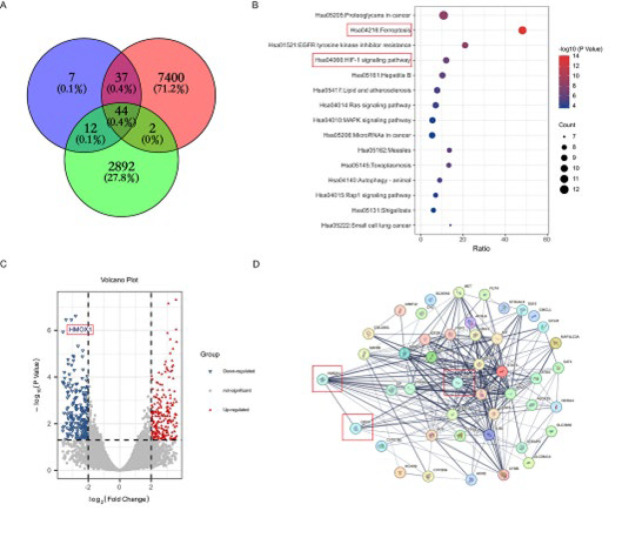
Network pharmacology procedures

A comprehensive screening of 100 drug target genes of EGCG was conducted using TCMSP. Through applying bioinformatics tools like GeneCard and DisGeNET, 7,483 genes linked to SCI were identified. An additional 2,950 genes were obtained from GEO (GSE92657), reflecting expression in an SCI model. The intersection of these datasets, determined using Venn analysis, revealed 44 common genes (Figure 2A). KEGG pathway enrichment analysis indicated significant involvement in the Ferroptosis and HIF-1 signaling pathways (Figure 2B). We used volcano plot analysis to examine differential gene expression and found that HMOX1 was significantly down-regulated (Figure 2C). To explore protein-protein interactions (PPI), networks were generated with the STRING database and analyzed in Cytoscape, identifying HMOX1, GPX-4, and HIF-1A as key hub genes based on their degree scores, which were above average (Figure 2D).

### EGCG attenuated spinal cord injury by inhibiting ferroptosis in vivo and in vitro

**Figure 3:**
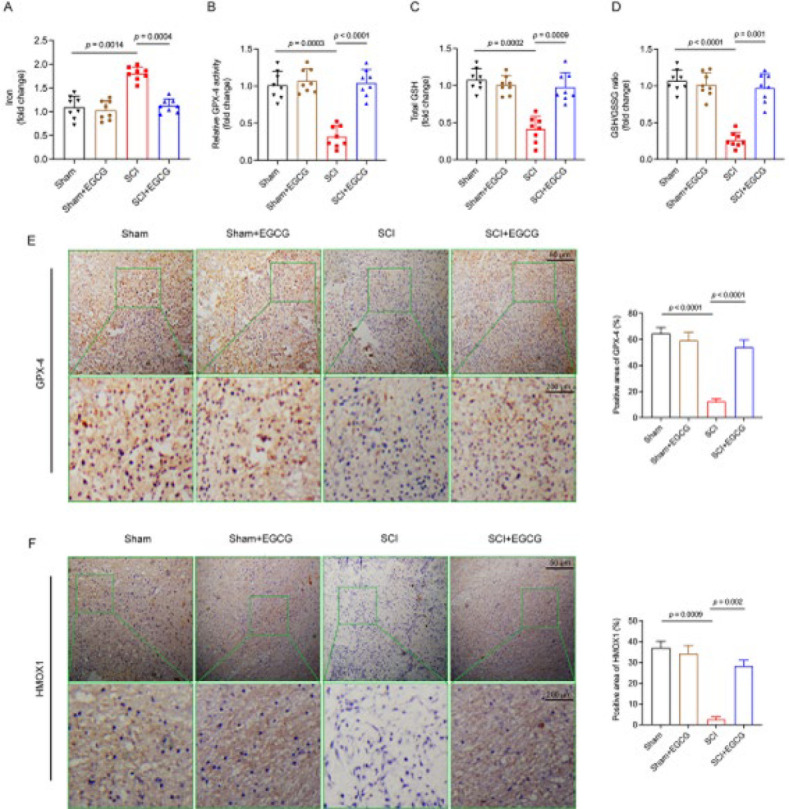
EGCG attenuates spinal cord injury by inhibiting ferroptosis* in vivo*

**Figure 4:**
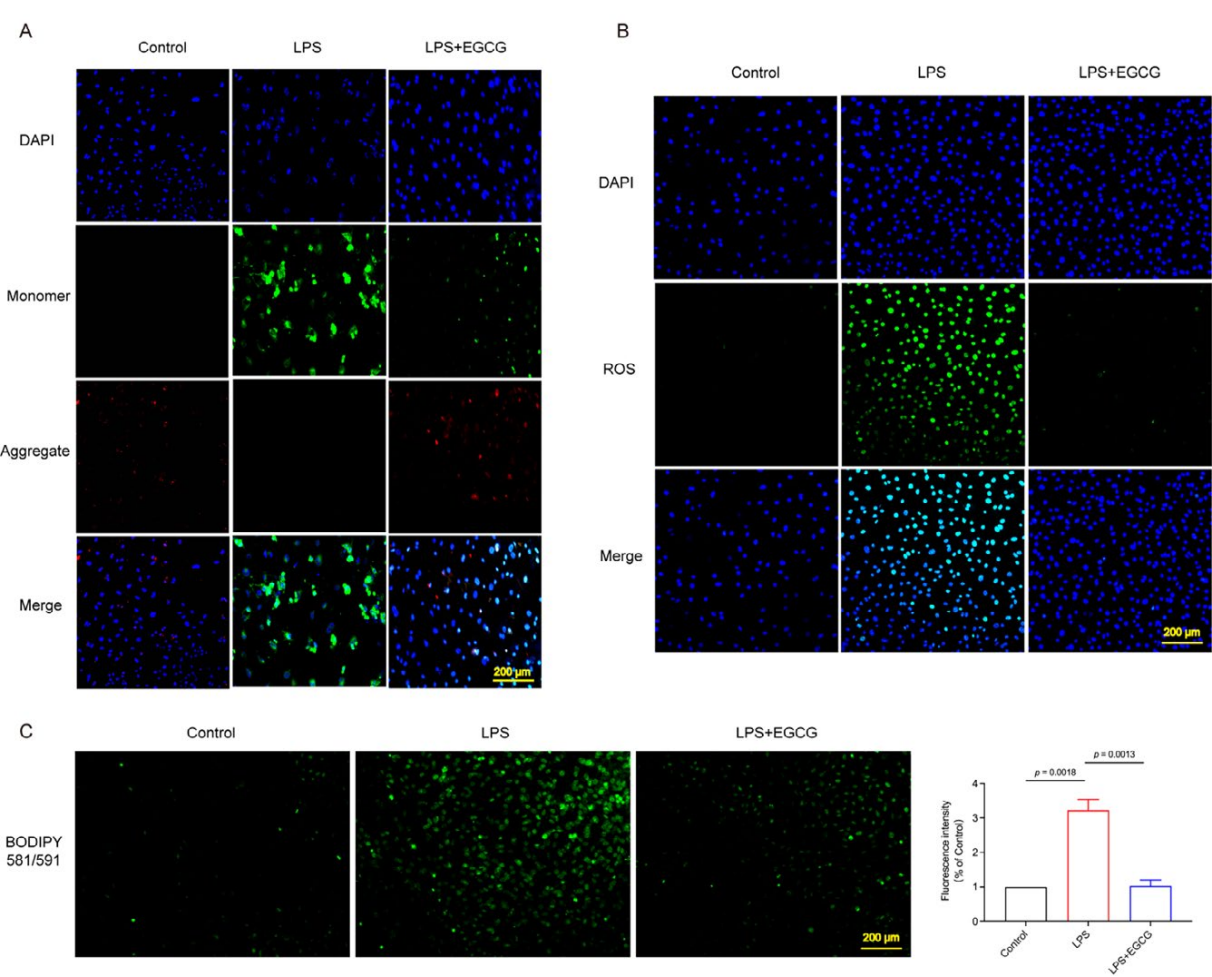
EGCG inhibits LPS-induced damage in HT22 cells

Using bioinformatics technology, we discovered a link between SCI and ferroptosis. Consequently, we measured iron levels, GPX-4 activity, GSH levels, and the GSH/GSSG ratio in spinal cord tissue at 12 weeks. Compared with the Sham group, iron levels were increased in the SCI group and significantly decreased in the SCI + EGCG group (Figure 3A). Furthermore, GPX-4 activity, GSH levels, and the GSH/GSSG ratio showed an opposite trend compared to iron levels (Figure 3B-D). Immunohistochemical results indicated that the expression of HMOX1 and GPX-4 proteins in the spinal cord was significantly decreased in the SCI group compared to the Sham group. However, the expression of both proteins was significantly up-regulated in the SCI + EGCG group (Figure 3E-F). Ferroptosis is a form of programmed cell death characterized by abnormal iron metabolism and lipid peroxidation associated with changes in mitochondrial structure and function. Therefore, we selected mitochondrial membrane potential (MMP) as the evaluation index. *In vitro* experiments, JC-1 staining is commonly used to assess mitochondrial membrane potential (ΔΨm). The results revealed that, compared to the Control, the red/green fluorescence intensity significantly decreased in the LPS group but was noticeably increased in the LPS + EGCG group (Figure 4A). Excessive ROS accumulation can trigger cell ferroptosis-related pathways through a series of cascade reactions (21, 22). Our study demonstrated that EGCG reduced ROS levels in LPS-treated neuronal cells, as detected by ROS assays (Figure 4B). Results from the BODIPY probe indicated that LPS significantly increased intracellular lipid ROS levels in HT22 cells, while EGCG treatment had markedly opposing effects (Figure 4C). Overall, the results demonstrate that EGCG inhibits ferroptosis and alleviates SCI, but the precise mechanism will need to be confirmed in our subsequent experiments.

### EGCG mitigated spinal cord injury by inhibiting ferroptosis through the activation of HMOX1 expression and suppression of the HIF-1 signaling pathway

**Figure 5:**
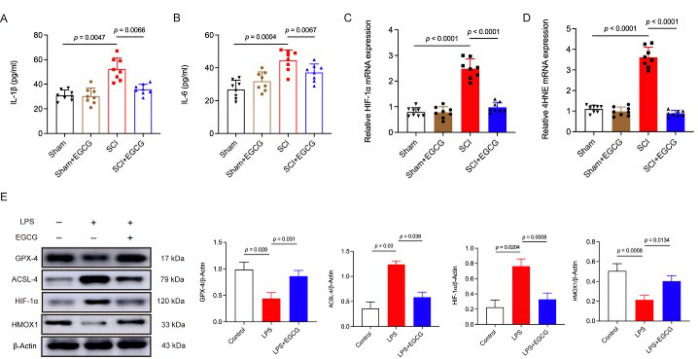
EGCG restrains HIF-1 signaling pathway activation

**Figure 6:**
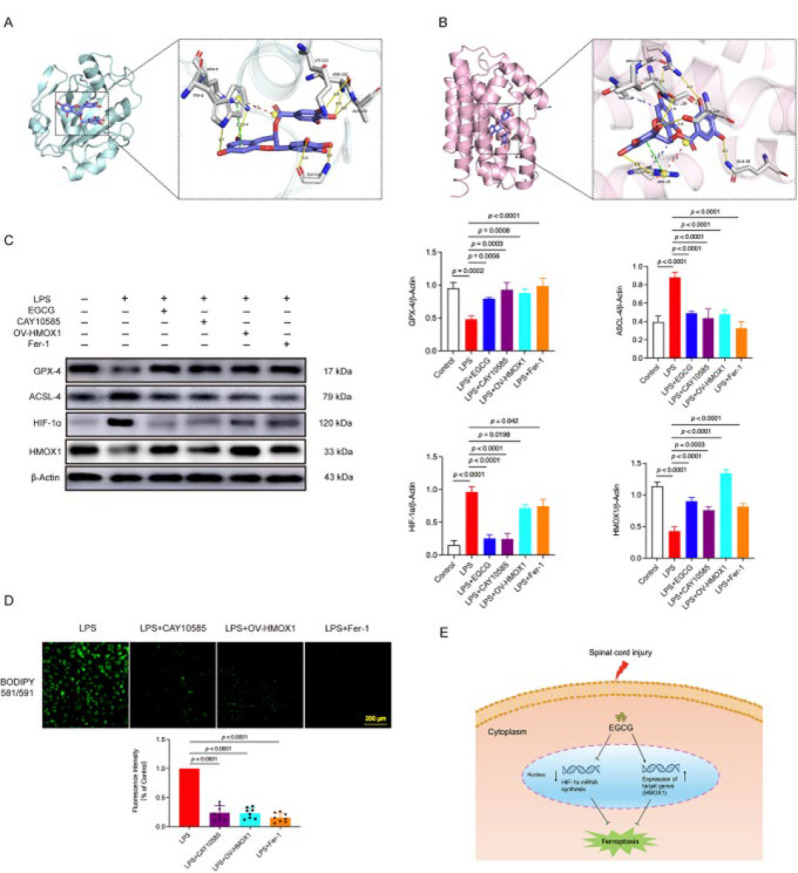
EGCG mitigated SCI by inhibiting ferroptosis via activation of HMOX1 expression and suppression of the HIF-1 signaling pathway

In an *in vivo* experiment, we detected the expression of inflammatory factors. Enzyme-linked immunosorbent assay results indicated that the levels of IL-1β and IL-6 were significantly elevated in the SCI mice. However, treatment with EGCG reduced the levels of these two pro-inflammatory cytokines (Figure 5A-B). qRT-PCR results revealed that mRNA expression levels of HIF-1α and 4HNE were up-regulated in the SCI group and down-regulated in the SCI + EGCG group (Figure 5C-D). Furthermore, western blot results showed that LPS up-regulated the expression of ACSL-4 and HIF-1α, while EGCG significantly down-regulated these proteins in LPS-treated HT22 cells. Conversely, the expression levels of GPX-4 and HMOX1 in each group exhibited the opposite trend (Figure 5E). Specific binding patterns of EGCG with target proteins were analyzed and optimized using PyMOL 2.3.0. Comprehensive analysis revealed that the docking binding energy of EGCG with HIF-1α was −8.8 kcal/mol, and with HMOX1 was −8.7 kcal/mol, indicating strong binding interactions with both proteins (Figure 6A-B). We introduced HIF-1 signaling pathway inhibitors (CAY10585), a lentiviral vector overexpressing HMOX1, and ferroptosis inhibitors *in vitro* to investigate the specific mechanism. Western blot results revealed that ACSL-4 and HIF-1α were significantly up-regulated, while GPX-4 and HMOX1 were down-regulated in the LPS group. However, the expression trends of these four proteins were reversed in the LPS+EGCG, LPS+CAY10585, LPS+OV-HMOX1, and LPS+Fer-1 groups (Figure 6C). Additionally, CAY10585, OV-HMOX1, and Fer-1 significantly reduced intracellular lipid ROS levels, as measured by BODIPY detection (Figure 6D).

## Discussion

The pathophysiology of SCI includes primary injury and secondary injury. The former is usually a mechanical injury to the spinal cord. The latter results from cellular and biological reactions to the primary injury involving the immune, nervous, and vascular systems, including hemorrhage, ischemia, oxidative stress, inflammatory response, neuronal cell death, demyelination, and scar formation (23). According to the time of injury, secondary injury can be divided into acute phase (within 48 hr), subacute phase (2~14 days), and chronic phase (after 14 days) (24). The acute phase is mainly manifested by spinal cord ischemia, vasogenic edema, and glutamate excitotoxicity. The subacute phase is mainly manifested by neuroinflammation, mitochondrial phosphorylation, reactive oxygen species (ROS), and reactive nitrogen species (RNS) generation. The chronic phase is mainly manifested by cell apoptosis and necrosis, axonal degeneration, axonal remyelination, axonal remodeling, and glial scar formation (2, 25). This study took the chronic stage of spinal cord injury as the research time point to explore the pathophysiological changes after nerve cells were damaged.

In recent years, a large number of experimental observations have shown that the active ingredients of TCM can inhibit the inflammatory response caused by SCI, reduce the release of inflammatory mediators, and inhibit the proliferation and activation of inflammatory cells, thereby reducing further damage to the spinal cord caused by inflammation (26, 27). Some active ingredients in TCM can stimulate nerve cells to release neurotrophic factors, such as nerve growth factor and brain-derived neurotrophic factor, promoting the regeneration and recovery of damaged neurons. Lipid peroxidation is another important pathological process caused by spinal cord injury. Some ingredients in TCM have anti-oxidant effects, inhibiting lipid peroxidation and reducing the damage of oxygen free radicals to the spinal cord (27). After spinal cord injury, microcirculation is affected due to vascular damage and inflammatory response, resulting in insufficient supply of nutrients and oxygen. Some ingredients in TCM can dilate blood vessels, reduce blood viscosity, and promote blood flow, thereby improving microcirculation in the SCI area and providing better nutrition and oxygen supply (28, 29). In addition, the active ingredients of some traditional Chinese medicines can inhibit cell apoptosis in the spinal cord injury area, increase the survival rate of damaged neurons, and promote their regeneration and connection, which helps to repair spinal cord function (30).

EGCG exhibits potent anti-oxidant properties that help prevent excessive autophagy or apoptosis, thereby mitigating ischemic injury in myocardial infarction (31). Recent studies indicate that EGCG reduces cellular lipid peroxidation and the expression of GPX4 and LC7A11 proteins in rats with gentamicin-induced kidney injury, demonstrating that EGCG can also inhibit iron deposition(32, 33). Therefore, in this study, we further investigated whether EGCG has a therapeutic effect on spinal cord injury. We selected experimental animals 28 days post-injury as the research model to further evaluate the therapeutic effect of EGCG on SCI model animals. The research results confirmed that EGCG effectively increased the body weight of SCI mice and promoted the recovery of hindlimb motor functions. Morphological analysis also revealed a significant reduction in spinal cord injury in the treated group, with the loss of white matter and central gray matter showing a tendency towards normal tissue. Additionally, the disorganization of nerve fibers was improved, and nerve cell necrosis was reduced. Subsequently, we used bioinformatics techniques to analyze key hub genes, and the results provided valuable insights into the direction of this study. Forty-four hub genes were identified, and KEGG enrichment analysis revealed that ferroptosis and the HIF-1 signaling pathway are closely associated with the mechanism by which EGCG treats SCI. Additionally, we identified HMOX1, GPX4, and HIF-1α as key functional proteins. These findings further guided our subsequent research into the underlying mechanisms.

Ferroptosis is a programmed cell death distinct from other forms, such as apoptosis, necrosis, and autophagy. It is characterized by the accumulation of iron-dependent lipid peroxides, which leads to oxidative damage and eventual cell death. GPX4 is a crucial enzyme that safeguards cells from ferroptosis by converting lipid hydroperoxides into non-toxic lipid alcohols. In the experiment, we observed a significant increase in iron levels and a decrease in GPX4 expression and GSH levels in SCI mice. Treatment with EGCG was able to suppress these changes. HMOX1 is an inducible enzyme recognized for its anti-inflammatory, anti-oxidant, and neuroprotective effects. Many studies have confirmed that HMOX1 is closely related to the occurrence of ferroptosis (34, 35). Our experimental results also showed that EGCG can increase the expression level of HMOX1, suggesting a certain connection between the two in terms of mechanism.

*In vitro* experiments are crucial for verifying the mechanisms underlying cellular processes. In LPS-treated HT22 cells, a significant decrease in mitochondrial membrane potential was observed, accompanied by a marked increase in ROS and lipid ROS levels. However, EGCG intervention demonstrated a significant inhibitory effect. The inflammatory response is also an important pathological process in SCI. ELISA analysis in SCI mice revealed increased levels of IL-1β and IL-6 in spinal cord tissue samples, along with significantly up-regulated mRNA expression of HIF-1α and 4HNE. Treatment with EGCG was able to reverse these changes. Similarly, in LPS-treated neuronal cells, the expression of ACSL-4 and HIF-1α was up-regulated, while the expression of GPX4 and HMOX1 was down-regulated. However, the effects of EGCG treatment were the opposite. The above results indicate that EGCG has the pharmacological effect of inhibiting inflammatory response and may be related to reducing the occurrence of ferroptosis.

Pathway-specific inhibitors and HMOX1 overexpression plasmids were introduced to validate the proposed mechanism further. As expected, CAY10585, OV-HMOX1, and Fer-1 suppressed the expression of ACSL-4 and HIF-1α while enhancing the expression of GPX4 and HMOX1, thereby inhibiting ferroptosis and the HIF-1 signaling pathway. Notably, EGCG produced similar effects. Furthermore, CAY10585, OV-HMOX1, and Fer-1 significantly alleviated lipid peroxidation damage. The above research analysis suggests that EGCG can alleviate spinal cord injury by up-regulating HMOX1 expression and inhibiting the HIF-1 signaling pathway, inhibiting ferroptosis.

While our *in vivo* and *in vitro* experiments supported the anticipated conclusions, certain limitations must be acknowledged. First, generalizing the model’s findings to clinical applications should be done with caution, as various factors within the human body must be considered. Second, although our results demonstrate that EGCG alleviated SCI in animal models, further investigations are needed to explore its effects in other models. Finally, more research is necessary to examine the interactions between EGCG and different cell types.

A C57BL/6 mouse model was used for the SCI study, with relevant indices measured 28 days post-injury. (A) Diagram of SCI and EGCG treatment, (B) Body weight was recorded at the indicated time points, (C) Footprint analysis of stride length, (D) Footprint analysis of stride width, (E) Quantification of swim test results by swimming scores, (F) H&E staining of spinal cord tissue section. *P<0.05 vs Sham group (n = 8); #*P*<0.05 vs SCI group (n = 8).

(A) Identifying the crossover target genes between EGCG and SCI through the use of a Venn Diagram, (B) KEGG enrichment analysis of the crossover target genes, (C) Volcano plot showing the differential protein expression, HMOX1 was down-regulated clearly, (D) Constructing the PPI network and screening for hub targets.

Spinal cord tissues were collected in 28 days to measure ferroptosis-related indicators. (A) Iron, (B) GPX-4 activity, (C) GSH level, (D) GSH/GSSG ratio, (E) Expression of GPX-4 was evaluated by immunohistochemistry analysis, (F) Expression of HMOX1 was evaluated by immunohistochemistry analysis.

(A) Mitochondrial membrane potential (Δψm) was measured using JC-1 staining, photos were taken using fluorescence microscopy. (B) Intracellular ROS level detection was determined by fluorescence microscopy, and a BODIPY probe was utilized for staining. (C) Intracellular lipid ROS.

Based on the results of the network pharmacology analysis, SCI is involved in the HIF-1 signaling pathway. (A) Expression level of IL-1β, (B) IL-6 expression level, (C) mRNA expression of HIF-1α, (D) mRNA expression of 4HNE, (E) protein expression levels of GPX-4, ACSL-4, HIF-1α, and HMOX1 associated with ferroptosis and HIF-1 signaling pathway.

(A) Interaction between protein HIF-1α and small molecule EGCG, (B) Interaction between protein HMOX1 and small molecule EGCG, (C) Protein expression levels of GPX-4, ACSL-4, HIF-1α and HMOX1, (D) Intracellular lipid ROS of HT22 cells was stained by BODIPY probe, (E) Schematic diagram showing the mechanism of EGCG alleviating spinal cord injury.

## Conclusion

Our study combined bioinformatics data analysis with experimental validation to reveal new insights into the therapeutic effects of EGCG in treating SCI. We found that EGCG mitigates spinal cord injury by inhibiting ferroptosis, primarily through activating HMOX1 expression and suppressing the HIF-1 signaling pathway. Although these findings highlight promising therapeutic potential, several limitations exist, including the lack of validation in clinical patients, diverse animal models, and other cell models. Further research is needed to explore this potential in greater detail.

## Declaration

We have not used any AI tools or technologies to prepare this manuscript.
